# Constant Electric and Magnetic Fields Effect on the Structuring and Thermomechanical and Thermophysical Properties of Nanocomposites Formed from Pectin–Cu^2+^–Polyethyleneimine Interpolyelectrolyte–Metal Complexes

**DOI:** 10.1186/s11671-015-1181-z

**Published:** 2015-12-10

**Authors:** V. Demchenko, V. Shtompel’, S. Riabov, E. Lysenkov

**Affiliations:** Institute of Macromolecular Chemistry, the National Academy of Sciences of Ukraine, 48 Kharkivske chaussee, Kyiv, 02160 Ukraine; V.O. Sukhomlyns’kyi Mykolayiv National University, 24, Nikolska Str., Mykolayiv, 54030 Ukraine

**Keywords:** Interpolyelectrolyte complexes, Interpolyelectrolyte–metal complexes, Nanocomposite, Structure, Thermomechanical properties, Thermophysical properties, Constant field

## Abstract

Applying wide-angle X-ray scattering method, thermomechanical analysis, and differential scanning calorimetry, the structural organization and properties of nanocomposites formed by chemical reduction of Сu^2+^ cations in the interpolyelectrolyte–metal complex (pectin–Cu^2+^–polyethyleneimine) under the influence of a constant magnetic and electric fields have been studied. It has been found that the chemical reduction of Cu^2+^ cations in the interpolyelectrolyte–metal complex bulk under constant electric and magnetic fields leads to formation of nanocomposite consisting of interpolyelectrolyte complex, including pectin–polyethyleneimine and nanoparticles of the metal Cu phase, whereas nanocomposite with Cu/Cu_2_O nanoparticles is formed in original state (without any field). It was observed that, under constant field, nanocomposites obtained have higher structural glass-transition temperatures and thermal stability.

## Background

In the last decade, considerable attention is paid to the scientific researches dealing with polymer nanocomposites, filled with nanoparticles of different metals or metal oxides [1–3].

Metallo-containing compounds can provide polymer materials with special optical, electrical, magnetic, and mechanical properties as well as catalytic activity [4–8]. The capability of functional groups on polyelectrolytes to bind metal ions offers a possibility for their application as sorbing agents, ion-exchange materials, components of selective membranes [8–10], or as precursors for preparation of polymer–inorganic hybrids via reduction or precipitation of metal ions [8–12]. Polymer–inorganic nanocomposites are important candidates for construction of photonic devices, band-pass filters, components of nonlinear optical systems, optical limiters, elements of microcircuit chips, etc. [5, 6, 13]. Polyelectrolyte-based materials, including ultrafine particles of silver and noble metals, exhibit antibacterial properties and are therefore promising objects for application in medicine [13–17].

The current methods of preparing polymer–metal nanocomposites are mainly multistage, for example, the synthesis of metal or metal oxide nanoparticles followed by their introduction into the polymer bulk [2, 3, 18]. The above approach has a disadvantage due to difficulties in providing a uniform nanoparticle distribution throughout the polymer matrix. An alternative method of nanocomposite synthesis is the reduction of metal ions (Мe^*n*+^) in interpolyelectrolyte–metal complexes formed via the introduction of metal salts into an interpolyelectrolyte complex based on two oppositely charged polyelectrolytes [8, 19, 20]. This method allows the preparation of nanocomposites with uniform distributions and controlled nanoparticle sizes in the polymer matrix.

The structuring and thermomechanical properties of pectin–Cu^2+^–polyethyleneimine interpolyelectrolyte–metal complexes (IMC) and related nanocomposites were studied previously [21]. It was found that the chemical reduction of Cu^2+^ cations in the IMC bulk with the use of NaBH_4_ results in interpolyelectrolyte complexes (IPEC) and Cu/Cu_2_O nanocomposites, and, at the molar ratio BH_4_^−^:Cu^2+^ = 6, the structure of the Cu metal phase manifests itself completely. As it was earlier shown [22], reduction of Cu^2+^ cations by NaBH_4_ in the triple polyelectrolyte–metal complexes, influencing by a constant magnetic field allows physico-mechanical properties of nanocomposites to be enhanced.

So, the aim of this work is to investigate the effect of constant electric and magnetic fields on the structural organization and thermomechanical and thermophysical properties of nanocomposites prepared involving a natural and synthetic polymers—pectin, polyethyleneimine, and Cu nanoparticles, formed from pectin–Cu^2+^–polyethyleneimine interpolyelectrolyte–metal complexes.

## Methods

To obtain the IPEC, the IMC, pectin–Cu^2+^–polyethyleneimine, and nanocomposites of IPEC–Cu/Cu_2_O or IPEC–Cu, the following reagents were used: anionic polyelectrolyte citrus pectin (Cargill Deutschland GmbH, Germany) with *М* = 3 × 10^4^, cationic polyelectrolyte anhydrous branched polyethyleneimine (PEI) (Aldrich) with *М*_n_ = 1 × 10^4^ and *М*_w_ = 2.5 × 10^4^, copper(II) sulfate pentahydrate (CuSO_4_ × 5H_2_O) (Aldrich) with *М* = 249.69, and sodium borohydride (NaBH_4_) (Aldrich) with *М* = 37.83.

IPEC samples were formed via mixing of 5 % aqueous solutions of pectin and PEI taken at a molar ratio of 1:1 at *Т* = 20 ± 2 °С. IPEC as films were prepared via pouring onto PTFE plates and drying up to constant masses at the same temperature. Dry IPEC films were washed in distilled water up to neutrality and dried repeatedly at 20 °С up to constant masses. The resulting films were 100-μm thick.

IMC samples were prepared via immersion of IPEC films into an aqueous solution of CuSO_4_ with a concentration of 0.1 mol/L at *Т* = 20 ± 2 °С for 24 h. The colorless IPEC films became dark blue.

The adsorption capacities of films, *А* (mmol/g), were calculated through the formula [23]

$$ A = \left({c}_{\mathrm{in}}\hbox{--}\ {c}_{\mathrm{eq}}\right)V/m, $$

where *m* is the mass of the adsorbent, *V* is the solution volume, and *c*_in_ and *c*_eq_ are the initial and the equilibrium concentrations of copper ions. For IMC films, *А =* 2.9 mmol/g.

The chemical reduction of Cu^2+^ cations in the IMC was conducted with NaBH_4_ (a molar ratio of BH_4_^−^:Cu^2+^ = 6.0) in an alkaline medium (pH 10.8) in a solvent mixture of water–isopropanol (4:1 vol.%) at *Т* = 20 ± 2 °С for 3 h (until the release of gaseous bubbles ceased). The concentration of NaBH_4_ in the aqueous alcohol solution was 0.1 mol/L. As a result of the reduction, IMC films changed color from blue to dark brown that confirms the formation of Cu_2_О nanoparticles in the polymer matrix [19].

The reduction of Cu^2+^ cations in the IMC was performed both in the absence and in the presence of a constant electric field (*E* = 1 × 10^6^ V/m) for 3 h between the plates of a plane capacitor and as well in constant magnetic field (*B* = 0.2 T) for 3 h between the poles of electromagnet. In both cases, film surface was placed perpendicularly to the field force lines (*Т* = 20 ± 2 °С). Values of the electric field intensity, magnetic field induction, and reduction time for Cu^2+^ cations were selected as optimal in our experiment.

The features of the amorphous and amorphous–crystalline structuring of the IPEC (pectin–PEI), the IMC (pectin–Cu^2+^–PEI), and nanocomposites of IPEC–Cu/Cu_2_O or IPEC–Cu were studied by wide-angle X-ray diffraction on a DRON-4-07 diffractometer (scientific-production company “Burevestnik,” Russia), whose X-ray optical scheme was used to “pass” primary-beam radiation through samples. X-ray diffraction studies were performed at *Т* = 20 ± 2 °С in Cu*К*_α_ radiation monochromated with a Ni-filter.

The size of the Cu/Cu_2_O nanoparticles and their distribution in the polymer matrix were examined with a JEM-1230 transmission electron microscope (JEOL, Japan) at a resolution of 0.2 nm.

Thermomechanical studies of polymer systems were conducted using the penetration method in the mode of a uniaxial constant load (*σ* = 0.5 MPa) on a UIP-70M device (central design engineering bureau of the special instrument making of the National Academy of Sciences of Russia). Linear heating of samples was performed at a rate of 2.5 °С/min in the temperature range from −100 to +350 °С.

Thermophysical researches were performed applying modulated differential scanning calorimetry (DSC) method on a DSC-2 installation (Perkin Elmer, Germany) modernized and equipped with the software IFA Gmb (Ulm). The measurements were carried out in a dry air environment in the temperature range from 20 to 330 °C. The heating rate was 2 °С/min.

## Results and Discussion

**Fig. 1:**
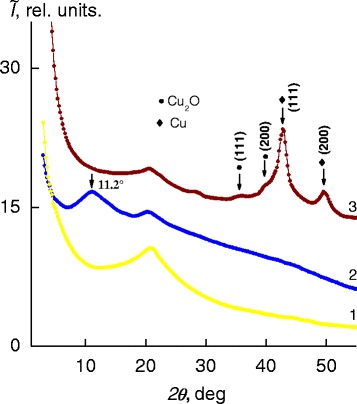
Wide-angle X-ray diffractograms of *1* the IPEC, *2* the IMC, and *3* the IPEC–Cu/Cu_2_O nanocomposite obtained via the chemical reduction of Cu^2+^ cations in the IMC at a molar ratio BH_4_^−^:Cu^2+^ = 6

The analysis of wide-angle X-ray diffractograms has shown that IPEC formed of pectin and PEI at a molar ratio of 1:1 is characterized by short-range ordering during translation of fragments of oppositely charged polyelectrolyte macromolecular chains in space. This circumstance is indicated by the appearance of one diffuse diffraction maximum with 2*θ*_*m*_ ~20.8° on the X-ray diffractogram of the IPEC sample (Fig. 1, curve *1*). The average value of the period of short-range ordering of fragments of complementary macromolecular chains of oppositely charged polyelectrolytes in the IPEC (the Bragg distance between the macromolecule chains of anionic and cationic polyelectrolytes in the IPEC) according to the Bragg equation is

$$ d=\lambda {\left(2{ \sin \uptheta}_m\right)}^{-1}, $$

where λ is the wavelength of the characteristic X-ray radiation, which is 4.3 Å (*λ* = 1.54 Å for Сu*K*_α_ radiation). Once the IMC is formed, the diffraction pattern changes. This is confirmed by the appearance of an intense diffuse diffraction maximum at 2*θ*_*m*_ ~11.2° (curve *2*) in the presence of a low-intensity amorphous halo, which, unlike that for the initial IPEC, has an angular position at 2*θ*_*m*_ ~20.4° (*d* ~4.4 Å). This diffraction maximum, according to [24], characterizes the existence of interpolyelectrolyte–metal complexes between the central ions (Cu^2+^) and ligands. Taking into account the angular position of this diffraction peak on the X-ray diffractogram of the IMC, average Bragg distance *d* between the macromolecule chains of polyelectrolytes coordinated with Cu^2+^ cations is found to be 7.9 Å.

With the use of the wide-angle X-ray scattering (WAXS) method, it was previously found [21] that the optimum molar ratio BH_4_^−^:Cu^2+^ for the reduction of Cu^2+^ cations by sodium borohydride in the IMC bulk followed by the formation of IPEC–Cu/Cu_2_O nanocomposites is 6. In the X-ray profile of these composites (curve *3*), the intense diffraction maximum at 2*θ*_m_ ~11.2° which is typical for the above polyelectrolyte–metal complexes is absent, unlike the two low-intensity maxima at 2*θ*_m_ ~35.6° and 40°, confirming formation of Сu_2_O particles in the IPEC bulk [25]. The X-ray profile additionally displays two intense maxima at 2*θ*_m_ ~42.8° and 49.6°, which are due to the structure of metallic copper.

Effective size *L* of Cu/Cu_2_O nanoparticles crystallites was found through the Scherrer method [26]:

$$ L=K\lambda {\left(\beta \cos {\theta}_m\right)}^{-1}, $$

where *К* is a constant related to the crystallite shape (for an unknown shape, К = 0.9) and *β* is the angular half-width (the width at half-height) of a diffraction maximum. It was shown that the average value of *L* is ~4.5 nm (for the calculation, diffraction maxima at 2*θ*_*m*_ = 42.8° and 49.6° (curve *3*) were used).

**Fig. 2:**
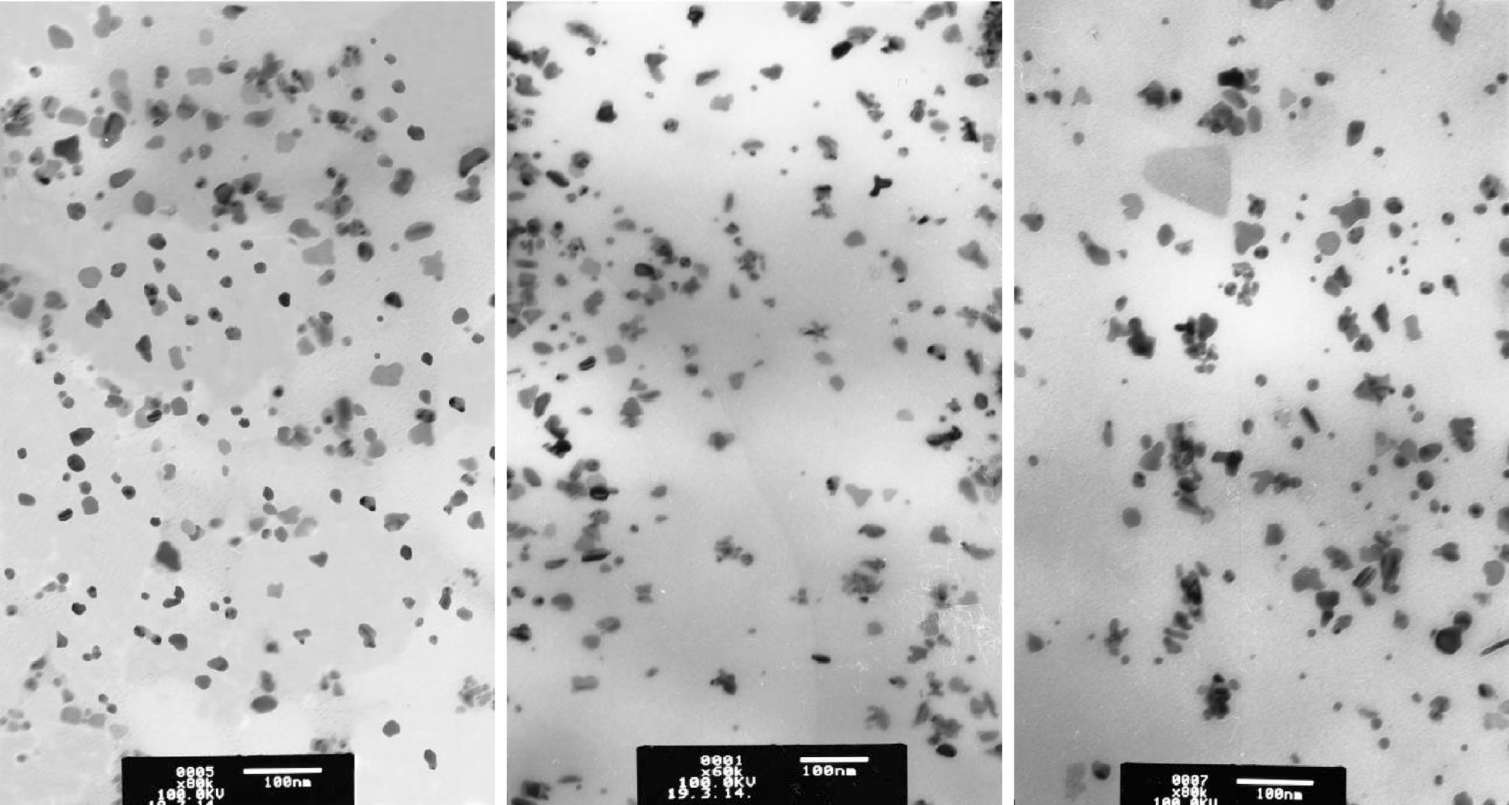
TEM micrograph of the IPEC–Cu/Cu_2_O nanocomposite obtained via the chemical reduction of Cu^2+^ cations in the IMC at a molar ratio BH_4_^−^:Cu^2+^ = 6

In addition, the formation of nanocomposites containing Cu/Cu_2_O nanoparticles from IMCs is corroborated by the data of transmission electron microscopy (TEM) (Fig. 2). The analysis of TEM images showed that the average size of Cu/Cu_2_O nanoparticles distributed randomly in the IPEC matrix is 10 nm; variation in sizes is ± 2 nm.

**Fig. 3:**
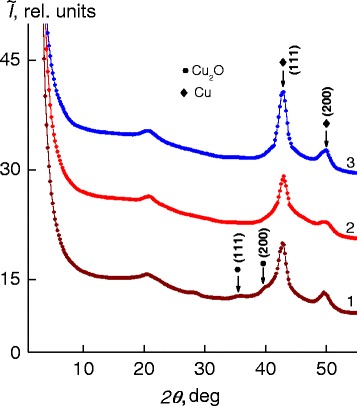
Wide-angle X-ray diffractograms of nanocomposites obtained in the initial state *1* on the base of the IPEC and Cu/Cu_2_O and on the base of the IPEC and Cu under *2* constant electric field and *3* constant magnetic field

Analyzing WAXS patterns of the initial nanocomposite and that formed under constant electric and magnetic fields, it was found that the chemical reduction of Cu^2+^ cations in the IMC bulk (at the molar ratio BH_4_^−^:Cu^2+^ = 6) under constant field results in nanocomposite of the IPEC and metal copper nanoparticles. This outcome is confirmed by the appearance of two diffuse diffraction maxima at 2*θ*_m_ ~43° and 50° and the absence of two maxima at 2*θ*_m_ ~35.6° and 40.0°, indicating presence of Сu_2_O nanoparticles in the IPEC bulk (Fig. 3, curves *1*–*3*).

In parallel, while studying constant electric and magnetic fields’ effect on the structural organization of nanocomposites, the constant fields’ influence on thermomechanical and thermophysical properties of nanocomposites was examined as well.

**Fig. 4:**
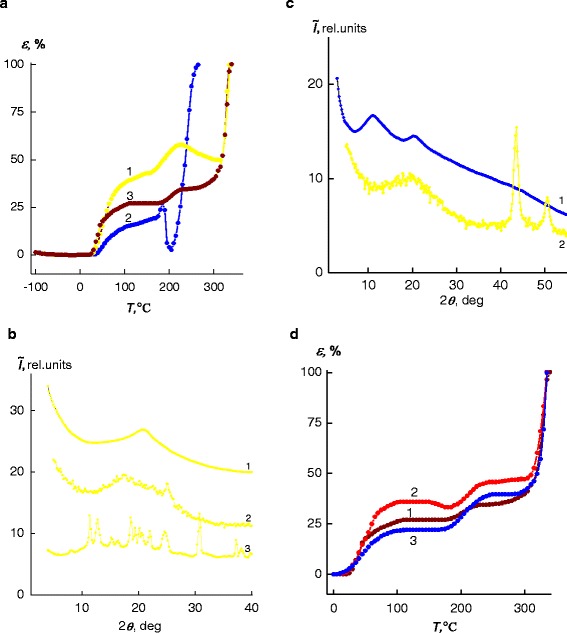
**a** Thermomechanical curves of *1* the IPEC, *2* the IMC, and *3* the IPEC–Cu/Cu_2_O nanocomposite obtained via the chemical reduction of Cu^2+^ cations in the IMC at a molar ratio BH_4_^−^:Cu^2+^ = 6. **b** Wide-angle X-ray diffractograms of IPEC based on pectin and PEI at *Т* = 20 ± 2 °С (*1*) and *Т* = 170 ± 2 °С (*2*) and pectin (powder) (*3*). **c** Wide-angle X-ray diffractograms of IMC at *Т* = 20 ± 2 °С (*1*) and *Т* = 190 ± 2 °С (*2*). **d** Thermomechanical curves of nanocomposites obtained via the chemical reduction of Cu^2+^ cations in the IMC at a molar ratio BH_4_^−^:Cu^2+^ = 6 in the initial state *1* on the base of the IPEC and Cu/Cu_2_O and on the base of the IPEC and Cu under *2* constant electric field and *3* constant magnetic field

Analysis of the thermomechanical IPEC’s curve (Fig. 4a, curve *1*) demonstrated temperature transitions that are associated with the temperatures of the glass transition and flow occur in the temperature ranges 25–145 °С and 265–350 °С, respectively. Furthermore, in the range of temperatures 150–245 °С, there is a temperature transition that is likely due to the melting of the crystallites of pectin in the IPEC [27]. Respectively, the strong deformational change has been observed in the melting process of pectin’s crystalline phase in IPEC [28]. This is also confirmed by the X-ray diffraction analysis data. Comparing IPEC wide-angle X-ray profiles at *Т* = 20 ± 2 °С and *Т* = 170 ± 2 °С (according to the thermomechanical analysis data, the pectin moiety’s melting temperature in the IPEC is 170 ± 2 °С) with profile of pure pectin (powder) (Fig. 4b, curves *1–3*), we can conclude that the following processes are taking place successively under the temperature growth IPEC: destruction of interpolyelectolyte complexes and crystallization of pectin fraction. These processes are accompanied by considerable diffraction peak’s displacement towards smaller scattering angels 2*θ* (from 20.8° to 17.4°), indicating the short-range ordering of IPEC parts—macromolecular chains’ fragments relating to both anion and cation polyelectrolytes, and, thus, resulting in the growth of the Bragg average distance between the macromolecules’ chains from 4.3 to 5.1 Å (Fig. 4b, curves *1, 2*). Also, one can see the diffraction maxima at 2*θ*_*m*_ = 25.0°, corresponding to pectin’s crystalline structure (Fig. 4b, curves *2*).

The formation of IMC leads to the appearance of a temperature transition at *T* = 205 °С on the thermomechanical curve, which seems to be due to melting of CuSO_4_ in the IMC bulk [29] that results in the transition of the polymer to the viscous-flow state (Fig. 4a, curve *2*). In its turn, Fig. 4c presents IMC profiles fixed at *T* = 20 ± 2 °С and *T* = 190 ± 2 °С.

In the IMC diffractogram monitored at *T* = 190 ± 2 °С, intensive diffraction peak at 2*θ*_*m*_ ≈ 11.2° (evidencing the presence of polyelectrolyte–metal complexes) is absent, but new peaks emerge at 2*θ*_*m*_ ≈ 43.5 and 50.5° that indicate the existence of copper’ crystalline structure [25]. Therefore, location of the IMC’s thermomechanical curve indicates that in the temperature area ranged from 170 to 205 °С (see Fig. 4a, curve *2*), the following successive processes occur in IMC sample: interpolyelectrolyte–metal complexes are destroyed, and then, the salt (CuSO_4_) transfers from its ionic form to the crystalline one and then melts.

Analysis of the thermomechanical curves of the IPEC, the IMC, and IPEC–Cu/Cu_2_O nanocomposite (see Fig. 4a) shows that during the transition from the IPEC to the IMC, glass-transition temperature *Т*_g_ increases, and while IMC is converting into the IPEC–Cu/Cu_2_O nanocomposite, *Т*_g_ significantly decreases (Table 1).

**Table 1:** Transition temperatures (data obtained from the thermomechanical analysis) for the polymer systems investigated

Type of system	*Т*_*g*_, °С	*Т*_*f*_, °С	*ε*, % (*Т* = 120 °С)
Pectin	60	–	20
Polyethyleneimine	−34.5	–	–
IPEC	53	319	40
IMC	57	205	16
IPEC–Cu/Cu_2_O	44	317	27
IPEC–Cu (electric field)	52	317	36
IPEC–Cu (magnetic field)	49	321	22

Simultaneously, while *T*_g_ is changed, the decrease of transition temperature to the viscous-flow state (*T*_f_) occurs in the following row:

$$ {T}_{f\left(\mathrm{I}\mathrm{PEC}\right)}>{T}_{f\left(\mathrm{I}\mathrm{PEC}\hbox{-} \mathrm{C}\mathrm{u}/{\mathrm{Cu}}_2\mathrm{O}\right)}>{T}_{f\left(\mathrm{I}\mathrm{M}\mathrm{C}\right)}. $$

Relative deformation value of polymer systems is seen in Table 1.

In addition, observing thermomechanical curves of the nanocomposites filled with Cu/Сu_2_O nanoparticles prepared without subjection to constant field and the IPEC–Cu nanocomposites formed under the influence of constant electric and magnetic fields (Fig. 4d), one can see that *Т*_g_ and *T*_f_ values are increased for the IPEC–Cu nanocomposite formed under electric and magnetic fields. This result indicates the higher thermal stability of this polymer system compared with its analog obtained without subjection to a field. At the same time, in nanocomposites affected by magnetic and electrical fields, anionic polyelectrolyte pectin’s melting of crystalline phase takes place in a wide temperature range between 170 and 250 °С, when nanocomposites without any field’s action reveal this interval at 180–230 °С (Fig. 4d). This effect is supposed to be due to formation more ordered structure in polymer matrix, when composites formed under impact of a physical field.

**Fig. 5:**
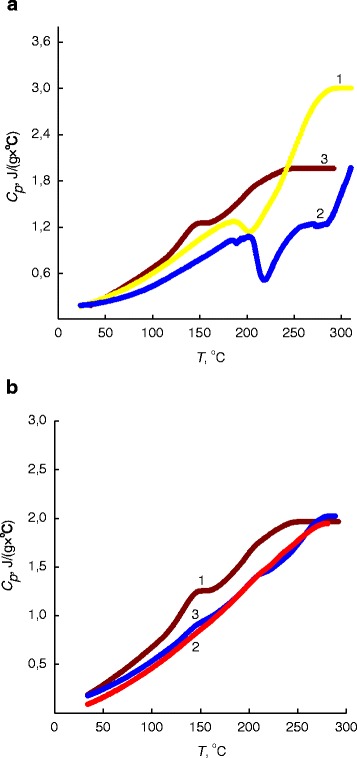
**a** Thermograms of *1* the IPEC, *2* the IMC, and *3* the IPEC–Cu/Cu_2_O nanocomposite obtained via the chemical reduction of Cu^2+^ cations in the IMC at a molar ratio BH_4_^−^:Cu^2+^ = 6. **b** Thermograms of nanocomposites obtained via the chemical reduction of Cu^2+^ cations in the IMC at a molar ratio BH_4_^−^:Cu^2+^ = 6 in the initial state *1* on the base of the IPEC and Cu/Cu_2_O and on the base of the IPEC and Cu under *2* constant electric field and *3* constant magnetic field

Analysis of the IPEC (pectin–PEI), IMC (pectin–Cu^2+^–PEI), and nanocomposites IPEC–Cu/Cu_2_O thermograms revealed that their transition temperatures are in a good agreement with those seen in the thermomechanical curves of these polymer systems (see Fig. 5a and Fig. 4a).

For IPEC at *T* = 185 °С and IMC at *T* = 202 °С, there are maximums, connected with pectin melting and CuSO_4_, correspondingly. Also, the minimums observed at *T* = 203 °С for IPEC and *T* = 218 °С for IMC could be explained by destructive processes proceeded in these systems (Fig. 5a, curves *1, 2*). In nanocomposite’s thermogram, there exist two maximums at *T* = 147 and 208 °С, corresponding to pectin’s crystallites melting, which have lower and higher melting temperatures, respectively (Fig. 5a, curves *3*).

In its turn, in thermograms of nanocomposites based on IPEC and Cu/Cu_2_O nanoparticles which were formed in original state (without any field) and those, having IPEC and Cu nanoparticles only, being formed under the action of constant electric and magnetic fields (Fig. 5b), one can observe that latter is characterized by higher *Т*_g_ and ∆*Т*_g_ values compared to systems prepared in the initial state (when a field is absent) (Table 2).

**Table 2:** Glass-transition temperature data (quantitative values) for the polymer systems according to DSC method

Type of system	*Т*_*g*_, °С	*∆Т*_*g*_, °С	*∆C*_*p*_, J/(g × °С)
IPEC	63	49	0.420
IMC	67	69.4	0.621
IPEC–Cu/Cu_2_O	60	47.5	0.378
IPEC–Cu (electric field)	67	88.8	0.860
IPEC–Cu (magnetic field)	61	69.8	0.363

## Conclusions

Constant electric and magnetic fields impact the structural organization and thermomechanical and thermophysical properties of nanocomposites based on the pectin–polyethyleneimine interpolyelectrolyte complex, and Cu nanoparticles formed from pectin–Cu^2+^–polyethyleneimine interpolyelectrolyte–metal complexes, involving sodium boron hydride as reducing agent, have been studied. It was found that the chemical reduction of Cu^2+^ cations in the IMC bulk under constant field proceeds with the formation of IPEC-based nanocomposite and nanoparticles of just metal Cu phase, whereas IPEC–Cu/Cu_2_O nanocomposite is formed in the absence of field.

Thermomechanical and thermophysical analyses reveal considerable changes in glass-transition temperatures in the following row—interpolyelectrolyte complex—interpolyelectrolyte–metal complex and nanocomposites based on interpolyelectrolyte complex–Cu/Cu_2_O. It has been shown that, under constant field, nanocomposites obtained on the base of interpolyelectrolyte complex–Cu have the higher structural glass-transition temperatures and thermal stability.
